# Prognostic value of red cell distribution width in patients undergoing percutaneous coronary intervention: a systematic review and meta-analysis

**DOI:** 10.3389/fcvm.2026.1787857

**Published:** 2026-07-22

**Authors:** Wanying Peng, Bingyan Deng, Jingya Wang, Mingsen Liu, Bokai Wan, Yining Yang, Xiaolin Yu

**Affiliations:** 1 Graduate School of Xinjiang Medical University, Urumqi, China; 2 Department of Cardiology, People’s Hospital of Xinjiang Uyghur Autonomous Region, Urumqi, China; 3 Xinjiang Key Laboratory of Cardiovascular Homeostasis and Regeneration Research, Urumqi, China

**Keywords:** coronary artery disease, meta-analysis, percutaneous coronary intervention, prognostic value of survival, red cell distribution width

## Abstract

**Background:**

Published studies have reported inconsistent findings regarding the association between preprocedural red cell distribution width (RDW) and adverse outcomes after percutaneous coronary intervention (PCI), and earlier reviews no longer reflect the currently available evidence. This updated systematic review and meta-analysis was conducted to reassess the association between preprocedural RDW and adverse outcomes in patients with coronary artery disease (CAD) undergoing PCI.

**Methods:**

PubMed, Embase, Web of Science, and the Cochrane Library were searched from inception to November 22, 2025. Standard major adverse cardiovascular events (MACE), all-cause mortality (ACM), and cardiovascular mortality (CVM) were prespecified as the primary outcomes, whereas PCI-specific stent-related outcomes were analyzed separately as exploratory outcomes. Hazard ratios (HRs) and odds ratios (ORs) with 95% confidence intervals (CIs) were extracted for quantitative synthesis when appropriate.

**Results:**

Twenty-four studies comprising 31 comparison groups and approximately 83,000 patients were included in the meta-analysis. Elevated preprocedural RDW was associated with higher risks of ACM (HR 1.46, 95% CI 1.31–1.63) and CVM (HR 1.66, 95% CI 1.33–2.07), with the most consistent association observed for ACM. The HR-based analysis of standard MACE showed a statistically significant association (HR 1.24, 95% CI 1.06–1.45; *p* = 0.007), but the finding was unstable in sensitivity analysis and should therefore be interpreted cautiously. After endpoint reclassification, only one study remained eligible for the OR-based analysis of standard MACE, precluding quantitative synthesis. In-stent restenosis (ISR) was analyzed separately as an exploratory stent-related outcome when eligible studies were available.

**Conclusions:**

Elevated preprocedural RDW was associated with adverse outcomes after PCI. The most consistent association was observed for ACM, whereas the evidence for CVM and standard MACE was less certain. PCI-specific stent-related outcomes should be interpreted as exploratory. Further prospective multicenter studies with standardized endpoint definitions and harmonized RDW cutoff values are warranted.

**Systematic Review Registration:**

https://www.crd.york.ac.uk/PROSPERO/view/CRD420251249457, PROSPERO, CRD420251249457.

## 1 Introduction

Despite substantial advances in revascularization strategies, patients with coronary artery disease (CAD) undergoing percutaneous coronary intervention (PCI) remain at considerable risk of adverse outcomes. This persistent residual risk underscores the need for simple, readily available, and clinically useful tools for prognostic stratification in this setting (1). Red cell distribution width (RDW), a routinely reported hematologic parameter that reflects variability in erythrocyte size, has attracted growing interest in cardiovascular research. Elevated RDW has been associated with inflammation, oxidative stress, impaired erythropoiesis, and adverse cardiovascular outcomes (2–5). However, current evidence suggests that RDW should be interpreted as a risk-related biomarker rather than as an established independent predictor. Nevertheless, the prognostic significance of RDW in patients undergoing PCI remains uncertain, as published studies have yielded inconsistent findings and differ in study design, patient characteristics, RDW cutoff values, and adjustment strategies (6–17). Recent studies have also explored RDW-based composite indices, such as the hemoglobin-to-RDW ratio, in both acute coronary syndrome and PCI populations, suggesting that combining RDW with other routinely available hematologic parameters may offer additional prognostic information (18, 19).

Although a previous meta-analysis suggested that elevated RDW was associated with adverse outcomes after PCI, that review was based primarily on studies published before 2019 and may no longer reflect the current evidence base (20). Several important issues also remain insufficiently addressed. First, a number of additional studies have been published in recent years, which may alter the overall evidence. Second, substantial between-study variability exists in RDW cutoff values, patient populations, and clinical presentations, including ACS, CCS, STEMI, NSTEMI, stable angina, and broader CAD cohorts, which may reduce the comparability and stability of pooled estimates. Third, effect estimates were not reported consistently across studies, as some studies provided multivariable-adjusted HRs or ORs whereas others reported less fully adjusted estimates, thereby limiting interpretability. Fourth, the definition of MACE was not uniform across studies, which may further affect the comparability of outcome assessment. To address these unresolved issues, we updated the evidence base, re-evaluated the associations of preprocedural RDW with adverse outcomes after PCI, and performed subgroup analyses according to population characteristics and RDW cutoff values to further explore potential sources of heterogeneity.

## 2 Materials and methods

### 2.1 Literature search

This study was conducted in accordance with the Preferred Reporting Items for Systematic Reviews and Meta-Analyses (PRISMA 2020) statement (21), and the study protocol was registered in the International Prospective Register of Systematic Reviews (PROSPERO; CRD420251249457). The search strategy was developed independently by two investigators (PWY and DBY). PubMed, Embase, Web of Science, and the Cochrane Library were systematically searched from database inception to November 22, 2025. A comprehensive search strategy incorporating terms related to red blood cell indices, erythrocyte indices, red cell distribution width (RDW), coronary artery disease (CAD), and percutaneous coronary intervention (PCI) was used. The detailed search strategy is provided in Supplementary Table S1.

### 2.2 Study selection

The bibliographies of relevant reviews and eligible studies were also manually screened to identify additional publications. No language restrictions were applied. Studies were considered eligible if they met the following criteria: (1) prospective or retrospective observational studies involving patients with coronary artery disease (CAD) who underwent PCI; (2) baseline red blood cell distribution width (RDW) evaluated as the exposure variable; (3) reporting at least one prespecified outcome of interest, including standard major adverse cardiovascular events (MACE), all-cause mortality (ACM), or cardiovascular mortality (CVM); (4) providing hazard ratios (HRs) or odds ratios (ORs) with corresponding 95% confidence intervals (CIs), or sufficient data for their calculation; (5) reporting extractable effect estimates for the association between RDW and the outcomes of interest; and (6) availability of the full-text article. Studies were excluded if they were: (1) reviews, editorials, conference abstracts, case reports, or correspondence articles; (2) lacking sufficient information to calculate effect estimates; (3) missing survival data or having a follow-up duration of less than 6 months; (4) not conducted in patients with CAD; or (5) based on duplicate or overlapping datasets. The prespecified primary outcomes were standard major adverse cardiovascular events (MACE), all-cause mortality (ACM), and cardiovascular mortality (CVM). Because MACE definitions varied across studies, only study-defined composite endpoints representing conventional cardiovascular outcomes, typically including death, repeat target vessel revascularization, and/or reinfarction, were considered eligible. PCI-specific stent-related outcomes, such as in-stent restenosis (ISR) and stent thrombosis, were analyzed separately as exploratory outcomes. A minimum follow-up duration of 6 months was required to ensure assessment of clinically relevant prognostic outcomes beyond the immediate peri-procedural period. Potentially overlapping cohorts were evaluated by comparing study centers, enrollment periods, patient populations, and author groups. When overlap was suspected, the study with the largest sample size, longest follow-up duration, or most complete outcome reporting was retained (Supplementary Table S3). Two investigators (PWY and DBY) independently screened titles and abstracts, reviewed full-text articles, and assessed study eligibility. Any disagreements were resolved through discussion and consensus.

### 2.3 Data extraction

Two investigators (PWY and DBY) independently extracted data using a predefined standardized form. The following information was collected: first author, publication year, study region, study design, sample size, population characteristics, follow-up duration, RDW cutoff value and its derivation method, timing of RDW assessment, original endpoint definitions, and effect estimates. Because no uniform RDW threshold was prespecified across studies, the original study-specific cutoff values and methods used to determine these thresholds were recorded whenever available. For studies in which RDW was analyzed as a continuous variable, the absence of a categorical cutoff value was documented and the study was not forced into a high-versus-low RDW classification framework. When both adjusted and unadjusted effect estimates were available, the most fully adjusted estimate was preferentially extracted. Studies that did not provide adjusted or independently interpretable RDW effect estimates were excluded from adjusted quantitative synthesis.

### 2.4 Quality assessment

The methodological quality of the included studies was assessed using the Newcastle–Ottawa Scale (NOS), which evaluates study selection, comparability, and outcome assessment. NOS scores range from 0 to 9 points, with higher scores indicating better methodological quality. All included comparison groups were independently evaluated by two investigators. Comparison groups with NOS scores ≥7 were considered to be of high methodological quality (7, 12, 22).

### 2.5 Statistical analysis

Hazard ratios (HRs), odds ratios (ORs), and corresponding 95% confidence intervals (CIs) were pooled to evaluate the prognostic association between RDW and adverse outcomes in patients with CAD undergoing PCI. Quantitative synthesis was performed only when at least two studies with sufficiently comparable outcome definitions were available. Standard MACE, ACM, and CVM were analyzed as primary outcomes. ISR was analyzed separately as an exploratory PCI-specific outcome when at least two eligible studies were available. Isolated stent thrombosis was summarized descriptively. Between-study heterogeneity was assessed using Cochran's *Q* test and the Higgins I^2^ statistic (23). A random-effects model was applied when I^2^ exceeded 50% or when significant heterogeneity was detected (*P* < 0.05). Otherwise, a fixed-effects model was used. Sensitivity analyses and subgroup analyses were conducted to evaluate the robustness of pooled estimates and to explore potential sources of heterogeneity. Because no uniform RDW cutoff value was prespecified, studies using categorical RDW definitions were analyzed according to their original study-specific thresholds. Studies analyzing RDW as a continuous variable were not forced into categorical subgroup classifications. Publication bias was assessed using funnel plots and Begg's or Egger's tests when appropriate. A two-sided *P*value < 0.05 was considered statistically significant. All statistical analyses were performed using STATA version 15.0 and Review Manager version 5.4.

## 3 Results

### 3.1 Study characteristics

**Figure 1:**
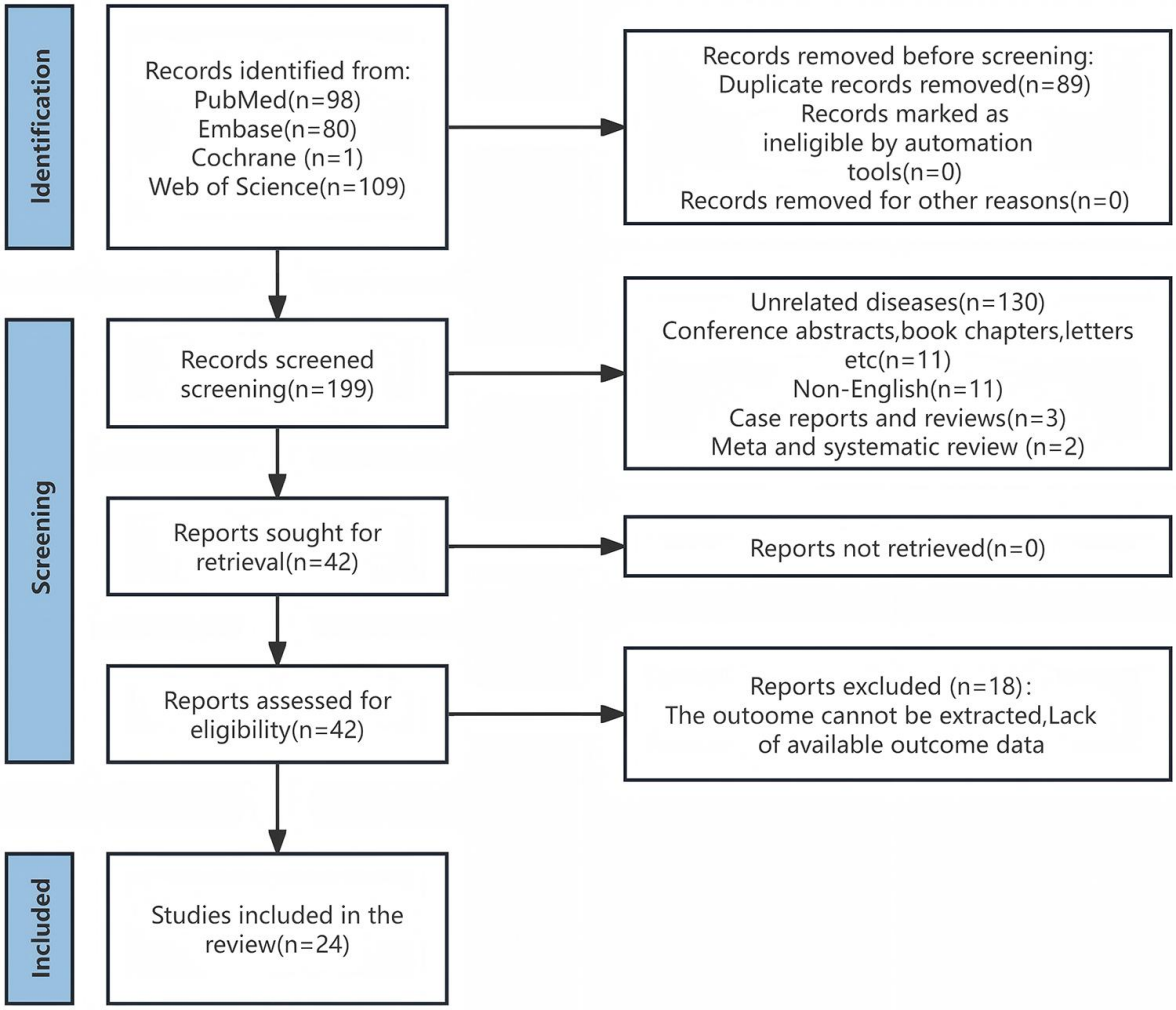
PRISMA 2020 flow diagram of study identification, screening, eligibility assessment, and inclusion.

A total of 288 records were initially identified through database searching, including 98 from PubMed, 80 from Embase, 1 from the Cochrane Library, and 109 from Web of Science. After removal of 89 duplicate records, 199 records were screened by title and abstract. Of these, 157 records were excluded, leaving 42 reports for full-text assessment. All 42 reports were retrieved and assessed for eligibility. Eighteen reports were excluded because outcome data could not be extracted or were insufficient for quantitative synthesis. Ultimately, 24 studies comprising 31 comparison groups and approximately 83,000 patients were included in the meta-analysis (Figure 1). Several studies contributed more than one independent comparison group because they reported separate analyses for different cohorts or outcome categories. Therefore, unless otherwise specified, subsequent quantitative analyses were conducted at the comparison-group level rather than the study level. Sample sizes ranged from 96 to 10,669 participants. Among the included studies, three enrolled patients with acute coronary syndrome (ACS), six focused on ST-segment elevation myocardial infarction (STEMI), two examined non-ST-segment elevation myocardial infarction (NSTEMI), one included patients with acute myocardial infarction and high thrombotic burden, one addressed chronic coronary syndrome (CCS), three investigated stable angina, and one focused on chronic total occlusion (CTO). The remaining seven studies included broader coronary artery disease (CAD) populations. The 24 studies were published between 2009 and 2025 and contributed 31 comparison groups in total. The included comparison groups were conducted in China (*n* = 9) (7–10, 13, 15, 22, 24, 25), Japan (*n* = 5) (26, 27), Turkey (*n* = 7) (6, 11, 14, 16, 28–30), the United States (*n* = 3) (31, 32), and Poland (*n* = 1) (33), whereas six comparison groups were derived from multicenter cohorts (12, 34, 35). Among these, 25 comparison groups were retrospective and 6 were prospective.

All studies were published in English, and the study periods ranged from 2002 to 2022. The mean or median age ranged from 55.8 to 68.1 years. All patients underwent PCI. Most studies evaluated RDW using study-specific categorical thresholds, whereas several studies analyzed RDW as a continuous variable; therefore, no uniform high-versus-low RDW definition was imposed across the included studies. RDW was measured before PCI in all 31 comparison groups. Based on endpoint reclassification according to the original study definitions, the included comparison groups were categorized into standard MACE, ACM, CVM, and PCI-specific stent-related outcomes. The latter included studies reporting ISR or isolated stent thrombosis, which were not treated as equivalent to standard MACE, only those with adjusted or independently interpretable RDW effect estimates were eligible for exploratory quantitative synthesis. After endpoint reclassification, the number of studies contributing to standard MACE, ACM, CVM, and exploratory stent-related outcomes was updated accordingly (Table 1). Unless otherwise specified, quantitative results in the following sections are reported at the level of comparison groups rather than individual studies.

**Table 1:** Basic characteristics of the included studies and comparison groups.

Author	study period	region	study design	Population	postoperative	No. of patients	Gender		Mean/median Age	RDW cut-off	Method for cut-off determination	follow-up months	MACE	MACEs definition	All-­cause mortality	Cardiovascular mortality	ISR/stent-related outcome
							Male	Female									
Tunçez, A. (16)	2009–2013	Turkey	Retrospective cohort	STEMI	preperative	321	276	45	NA	14.2	primary analysis used study-specific tertiles; ROC curve analysis	16.9 ± 8.7	NA	NA	NA	NA	OR:1.397, 95%CI:1.177-1.657
Karataş, M. B. (29)	2009–2013	Turkey	Retrospective cohort	STEMI	preperative	621	464	157	NA	13.4	ROC curve analysis	22	NA	NA	HR:1.24, 95% CI: 1.03-1.47	NA	NA
Huo, L. K. (24)	2019–2022	China	Retrospective cohort	CAD	preperative	4651	2967	1684	NA	NA	spline-based reported cutoff	14.3	NA	NA	HR: 1.437, 95%CI1.346-1.535	NA	NA
Li, C. (15)	2010–2013	China	Retrospective cohort	CTO	preperative	416	316	100	59.8 ± 11.5	NA	not specified; RDW analyzed as a continuous variable	14.4 ± 3.3	NA	NA	NA	NA	OR:1.324, 95%CI:1.134-1.545
Isik, T. (15)	2010-2010	Turkey	Prospective cohort	STEMI	preperative	96	74	22	60.6 ± 12.5	13.85	ROC curve analysis	48	HR: 5.26， 95% CI: 1.71-6.1	MACE were defined as cardiovascular mortality, repeat target vessel revascularization (TVR), and reinfarction (re-MI).no stroke	NA	NA	NA
Tsuboi, S. (27)	2000–2007	Japan	Retrospective cohort	Stable CAD	preperative	560	448	112	66.6	13.1	cohort median-based grouping	46.8	NA	NA	HR:2.56; 95%CI:1.12-6.62	HR:16.2; 95% CI,:2.09-124.7	NA
Liu, X. M. (22)	2009–2011	China	Retrospective cohort	NSTEACS	preperative	2,585	1,983	602	68.1	12.2	cohort median-based grouping	18	NA	NA	HR:2.171, 95%:CI:1.007-4.680	NA	NA
Zhuo, M. F. (13)	2018–2020	China	Retrospective cohort	acute myocardial infarction with high throm botic load	preperative	164	88	76	64.8	NA	not reported; RDW analyzed as a continuous variable	12	OR1.781, 95%CI:1.157-2.741	recurrent angina pectoris + recurrent acute MI + sudden cardiac death + malignant arrhythmia + congestive heart failure + other cardiovascular events	NA	NA	NA
Zhou, J. (12)	2016–2018	two centers	Retrospective cohort	ACS	preperative	215	61	154	NA	NA	not reported; RDW analyzed as a continuous variable	36	NA	NA	NA	NA	OR:1.54, 95%CI:1.14-2.08
Yildiz, A. (11)	2008–2013	Turkey	Retrospective cohort	CAD	preperative	269	203	66	62 ± 11	NA	not reported; RDW analyzed as a continuous variable	12	NA	NA	NA	NA	OR:1.4, 95%CI:1.18-1.67
Yao, H. M. (10)	2009–2011	China	Prospective cohort	CAD	preperative	2,169	1,468	701	60.2 ± 10.9	13	cohort quartile-based grouping	29	HR：1.21; 95% CI：1.04-1.39	death + MI + stroke	HR:1.37; 95% CI: 1.15-1.62	NA	NA
Wu, T. T. (9)	2008–2016	China	Retrospective cohort	CAD	preperative	6,050	4,495	1,555	NA	13.1	cohort median-based grouping	35.9 ± 22.6	HR：1.155; 95% CI0.994-1.341	cardiac death + recurrent MI + TVR	HR:1.203; 95%CI:0.941-1.57	HR:1.331; 95% CI:1.009-1.755	NA
Poludasu, S. (32)	2003–2005	United States	Retrospective cohort	STEMI	preperative	859	424	435	NA	13.3	empirically determined cut points based on hazard-ratio differences across RDW deciles	48	NA	NA	HR:1.23; 95%CI: 0.51-2.96	NA	NA
Poludasu, S. (32)	2003–2005	United States	Retrospective cohort	STEMI	preperative	859	424	435	NA	15.7	empirically determined cut points based on hazard-ratio differences across RDW deciles	48	NA	NA	HR:8.65; 95%CI:2.82-26.56	NA	NA
Liao, M. T. (35)	2006–2017	multi-center	Retrospective cohort	CAD	preperative	10,669	8,382	2,287	65.4 ± 12.1	13.4	ROC curve analysis	12	NA	NA	HR:1.394; 95%CI1.078-1.804	HR:1.533; 95% CI 1.049-2.240	NA
Liao, M. T. (35)	2006–2017	multi-center	Retrospective cohort	CAD	preperative	10,669	8,382	2,287	65.4 ± 12.1	14.1	ROC curve analysis	12	NA	NA	HR:1.592; 95%CI1.208-2.099	HR:1.568; 95% CI 1.029-2.387	NA
Liao, M. T. (35)	2006–2017	multi-center	Retrospective cohort	CAD	preperative	10,669	8,382	2,287	65.4 ± 12.1	14.8	ROC curve analysis	12	NA	NA	HR:2.003; 95%CI1.518-2.643	HR:1.609; 95% CI 1.031-2.509	NA
Liao, M. T. (35)	2006–2017	multi-center	Retrospective cohort	CAD	preperative	10,669	8,382	2,287	65.4 ± 12.1	15.9	ROC curve analysis	12	NA	NA	HR:2.689; 95%CI2.076-3.485	HR:2.710; 95% CI:1.825-4.026	NA
Uyarel, H. (30)	2003–2008	Turkey	Retrospective cohort	STEMI	preperative	2506	2075	431	56.6 ± 11.8	14.8	laboratory upper limit of normal	21	NA	cardiovascular mortality + reinfarctio*n* + repeat target-vessel revascularization (PCI or surgery)	NA	HR:2.703; 95%CI:1.208-6.048	NA
Fatemi, O. Paranilam, J.(34)	2004–2007	four centers	Retrospective cohort	CAD	preperative	1,435	1,123	3,12	65 ± 11.8	NA	center-normalized quartile stratification	12	NA	NA	HR:1.03; 95%CI:0.88-1.21	HR:1.65,; 95%CI:1.22-2.23	NA
Osadnik, T. (33)	2007–2011	Poland	Retrospective cohort	Stable coronaryartery disease	preperative	2,535	1,799	736	NA	13.6	cohort quartile-based grouping	30	NA	NA	HR:1.23; 95% CI:1.13-1.35	NA	NA
Moriya, S. (26)	2002–2016	Japan	Prospective cohort	CCS	preperative	1,827	1,576	251	64.9 ± 9.6	12.7	cohort quartile-based grouping	74.4	NA	NA	HR:1.46; 95%CI:1.24-1.69	NA	NA
Moriya, S. (26)	2002–2016	Japan	Prospective cohort	CCS	preperative	1,827	1,576	251	64.9 ± 9.6	12.7	cohort quartile-based grouping	74.4	NA	NA	HR:1.14; 95%CI:0.57-2.27	NA	NA
Moriya, S. (26)	2002–2016	Japan	Prospective cohort	CCS	preperative	1,827	1,576	251	64.9 ± 9.6	12.7	cohort quartile-based grouping	74.4	NA	NA	HR:1.62; 95%CI:0.85-3.11	NA	NA
Moriya, S. (26)	2002–2016	Japan	Prospective cohort	CCS	preperative	1,827	1,576	251	64.9 ± 9.6	12.7	cohort quartile-based grouping	74.4	NA	NA	HR:1.95; 95%CI:1.04-3.67	NA	NA
Liu, X. M. (25)	2009–2011	China	Retrospective cohort	≥65 years’ CAD	preperative	1,891	1,252	639	NA	12.3	cohort median-based grouping	17.6	NA	NA	HR:2.301; 95%CI:1.106-4.785	NA	NA
Pan, R. R. (8)	2016–2018	China	Retrospective cohort	ACS	preperative	396	301	95	62.45 ± 11.28	28.83	ROC curve analysis	12	HR:1.258; 95%CI:1.066-1.363	cardiac death + recurrent MI + CHF + atherosclerotic heart disease + hypoxic encephalopathy + fatal/nonfatal ischemic stroke	NA	NA	NA
Zhao, K. (7)	2010–2014	China	Retrospective cohort	stable angina pectoris (SAP)	preperative	293	230	63	NA	NA	not reported; RDW analyzed as a continuous variable	8	NA	NA	NA	NA	OR:1.4; 95%CI:1.18-1.67
Ösken, A. (6)	2015–2017	Turkey	Retrospective cohort	ACS	preperative	903	623	280	55.8 ± 10.2	NA	not reported; RDW analyzed as a continuous variable	13	NA	NA	NA	NA	OR:1.183; 95%CI:1.034-1.353
Azab, B. (31)	2004–2006	United States	Retrospective cohort	NSTEMI	preperative	619	417	202	NA	14	cohort tertile-based grouping	48	NA	NA	HR:1.104; 95%CI:1.004-1.23	NA	NA
Çiçek, G. (28)	2010–2015	Turkey	Retrospective cohort	STEMI	preperative	2,603	2,129	474	57.6 ± 11.8	NA	not reported; RDW analyzed as a continuous variable	12	NA	cardiovascular death + re-infarctio*n* + TVR	NA	HR:1.213; 95%CI:1.138-1.291	NA

### 3.2 Study quality

The NOS scores of all 31 comparison groups ranged from 6 to 7, indicating overall moderate-to-high methodological quality (Supplementary Table S2).

### 3.3 Meta-analysis results

#### 3.3.1 RDW and MACE

**Figure 2:**
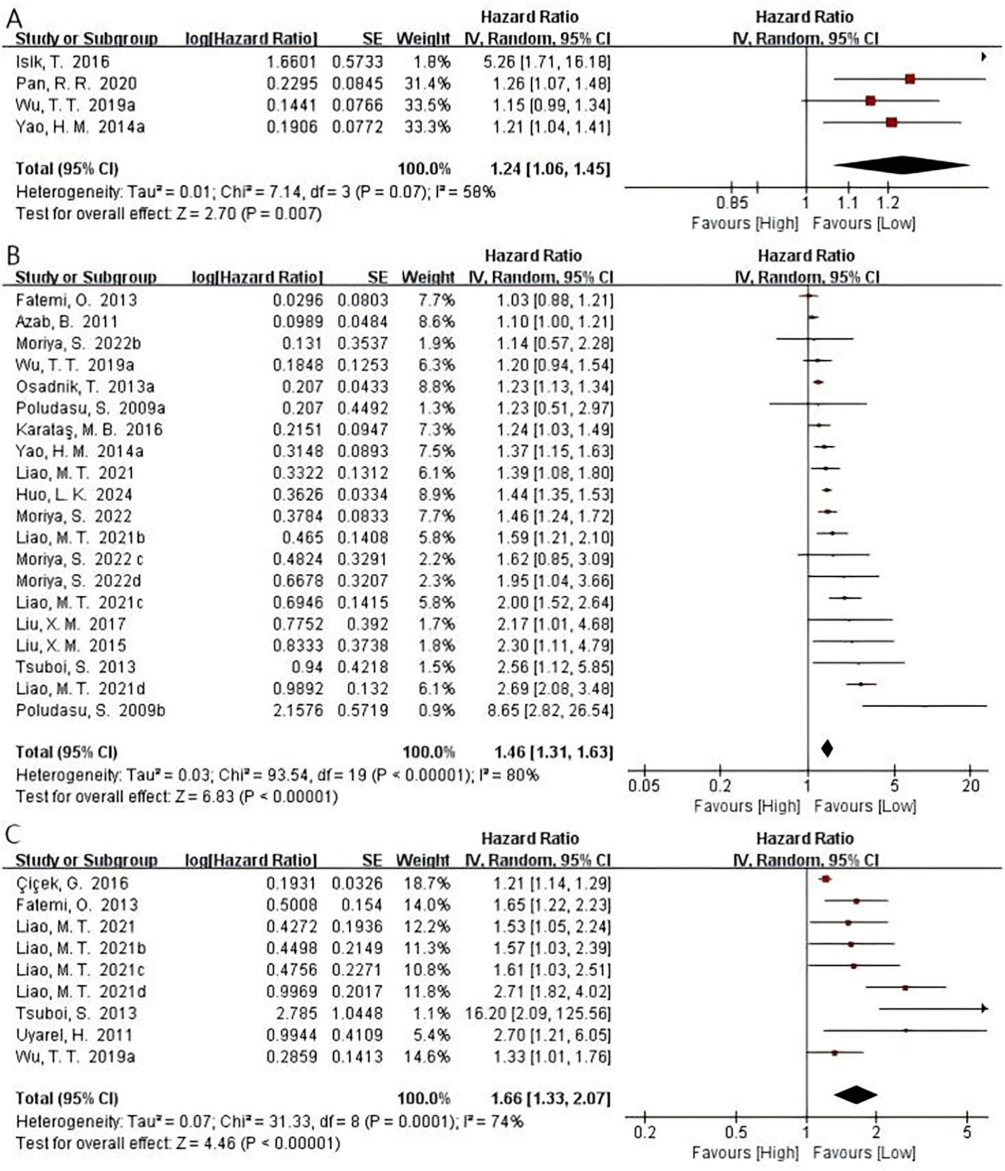
Forest plots for the associations of preprocedural red cell distribution width (RDW) with **(A)** standard major adverse cardiovascular events (MACE; HR-based analysis), **(B)** all-cause mortality (ACM), and **(C)** cardiovascular mortality (CVM).

After endpoint reclassification according to the original study definitions, only one study remained eligible for the OR-based analysis of standard MACE. Therefore, no quantitative synthesis was performed for standard MACE (OR), and the result was summarized narratively. By contrast, four comparison groups remained eligible for quantitative synthesis in the HR-based analysis of standard MACE because they reported broadly comparable composite cardiovascular endpoints (8–10, 14), A random-effects model was used because of substantial heterogeneity. The pooled estimate showed a statistically significant association between elevated RDW and standard MACE (HR 1.24, 95% CI 1.06–1.45; *p* = 0.007) (Figure 2A), with moderate between-study heterogeneity (I^2^ = 58%). However, this association was unstable in sensitivity analysis and should therefore be interpreted cautiously. Exploratory subgroup analyses were performed to investigate potential sources of heterogeneity; however, given the limited number of comparison groups and the instability observed in sensitivity analysis, these findings should be interpreted as exploratory rather than definitive.

#### 3.3.2 RDW and ACM

Data on RDW and all-cause mortality (ACM) were available from 20 comparison groups, comprising 74,818 patients (9, 10, 22, 24–27, 29, 31–35). Because of substantial heterogeneity, a random-effects model was applied. Elevated preprocedural RDW was associated with a higher risk of ACM (HR 1.46, 95% CI 1.31–1.63; *p* < 0.00001) (Figure 2B). Among the prespecified primary outcomes, ACM represented the most consistent association observed in the present meta-analysis. Subgroup analyses showed significant associations across all strata (Table 2). Heterogeneity analyses suggested that study design, RDW cutoff values, and regional distribution were the main sources of between-study heterogeneity.

**Table 2:** Pooled HRs for standard major adverse cardiovascular events, all-cause mortality (ACM), and cardiovascular mortality (CVM) in subgroup analyses.

Subgroup	MACE				For all-­cause mortalit				Cardiovascular mortality			
	Study groups	HR [95%CI]	*P* value	*I*2	Study groups	HR [95%CI]	*P* value	*I*2	Study groups	HR [95%CI]	*P* value	*I*2
Total	4	1.24 [1.06, 1.45]	0.007	58%	20	1.46 [1.31, 1.63]	＜0.00001	80%	9	1.66 [1.33, 2.07]	＜0.00001	74%
Study design												
Prospective	2	2.26 [0.54, 9.39]	0.26	85%	5	1.43 [1.28, 1.60]	＜0.00001	0%	NA	NA	NA	NA
Retrospective	2	1.20 [1.07, 1.34]	0.001	0%	15	1.48 [1.29, 1.68]	＜0.00001	84%	NA	NA	NA	NA
No. of patients												
≥900	2	1.18 [1.06, 1.31]	0.002	0%	15	1.48 [1.32, 1.67]	＜0.00001	77%	8	1.60 [1.30, 1.97]	＜0.00001	72%
＜900	2	2.30 [0.58, 9.18]	0.24	84%	5	1.46 [1.06, 2.01]	0.02	77%	1	16.20 [2.09, 125.56]	0.008	NA
Mean/median Age												
≥60.4 y	2	2.30 [0.58, 9.18]	0.24	84%	11	1.65 [1.33, 2.05]	＜0.00001	80%	6	1.71 [1.36, 2.16]	＜0.00001	55%
＜60.4 y	1	1.21 [1.04, 1.41]	0.01	NA	1	1.37 [1.15, 1.63]	0.0004	NA	2	1.63 [0.76, 3.48]	0.21	74%
RDW cut-off												
≥13.2	2	2.30 [0.58, 9.18]	0.24	84%	9	1.56 [1.28, 1.90]	＜0.00001	88%	5	1.87 [1.45, 2.42]	＜0.00001	37%
＜13.2	2	1.18 [1.06, 1.31]	0.002	5%	9	1.43 [1.28, 1.59]	＜0.00001	4%	2	3.75 [0.34, 41.84]	0.28	82%
Follow-up												
≥30 months	2	0.42 [0.16-1.12]	0.08	0%	10	1.34 [1.16, 1.55]	0.0001	66%	2	3.75 [0.34, 41.84]	0.28	82%
＜30 months	2	2.22 [0.51, 9.65]	0.29	85%	10	1.54 [1.32, 1.81]	＜0.00001	82%	7	1.68 [1.31, 2.15]	＜0.0001	76%
Region												
Asia	3	1.20 [1.10, 1.32]	＜0.0001	0%	11	1.41 [1.33, 1.50]	＜0.00001	5%	2	3.7[0.34,41.84]	0.28	82%
Non-Asia	1	5.26 [1.71, 16.18]	0.004	NA	4	1.25 [1.01, 1.56]	0.04	80%	2	1.63 [0.76, 3.48]	0.21	74%
multi-center	NA	NA	NA	NA	5	1.64 [1.15, 2.35]	0.007	91%	5	1.76 [1.44, 2.16]	＜0.00001	30%

#### 3.3.3 RDW and CVM

Nine comparison groups involving 55,830 patients evaluated the association between RDW and cardiovascular mortality (CVM) (9, 27, 28, 30, 34, 35). Because of substantial heterogeneity, a random-effects model was used. Elevated preprocedural RDW was associated with a higher risk of CVM (HR 1.66, 95% CI 1.33–2.07; *p* < 0.00001) (Figure 2C). Exploratory subgroup analyses for CVM showed variability across strata, but several subgroup estimates were based on small numbers of comparison groups and should therefore be interpreted cautiously. The results of these subgroup analyses are summarized in Table 2 and are presented as exploratory findings rather than definitive evidence. Heterogeneity analyses suggested that RDW cutoff values and regional distribution were the main contributors to between-study heterogeneity.

#### 3.3.4 RDW and ISR (exploratory analysis)

Studies reporting in-stent restenosis (ISR) were analyzed separately from the primary endpoints because ISR represents a PCI-specific stent-related outcome rather than a standard composite MACE endpoint. Eligible ISR studies were pooled in an exploratory OR-based analysis only when adjusted or independently interpretable RDW effect estimates were available. Based on these criteria, three eligible comparison groups were included in the quantitative synthesis (7, 11, 12). The exploratory pooled analysis showed that elevated RDW was associated with a higher risk of ISR (OR=4.13, 95% CI:3.57–4.77, *p* < 0.00001), with no significant between-study heterogeneity (I^2^ = 0%; Supplementary Figure S1). Li et al. 2019 and Ösken et al. 2024 (6, 15) were retained in the review as studies reporting ISR, but were not entered into the exploratory pooled ISR analysis because adjusted independent RDW effect estimates were not available. In Li et al. 2019, RDW was analyzed as a continuous variable without a study-defined categorical threshold, whereas Ösken et al. 2024 primarily focused on the systemic immune-inflammation index and did not provide an independently interpretable adjusted effect estimate for RDW suitable for quantitative synthesis. Sensitivity analysis demonstrated that the pooled association remained statistically significant after sequential omission of each study, with all recalculated 95% confidence intervals remaining above 1, supporting the relative robustness of this exploratory finding (Supplementary Figure S2). Because of the small number of included studies, publication bias testing and subgroup analyses were not performed. One study reported stent thrombosis as an isolated stent-related outcome and was summarized descriptively rather than quantitatively pooled. Because only three eligible comparison groups were available and ISR was prespecified as an exploratory PCI-specific outcome, the corresponding forest plot and sensitivity analysis were presented in the Supplementary Materials rather than as primary figures.

### 3.4 Sensitivity analysis

**Figure 3:**
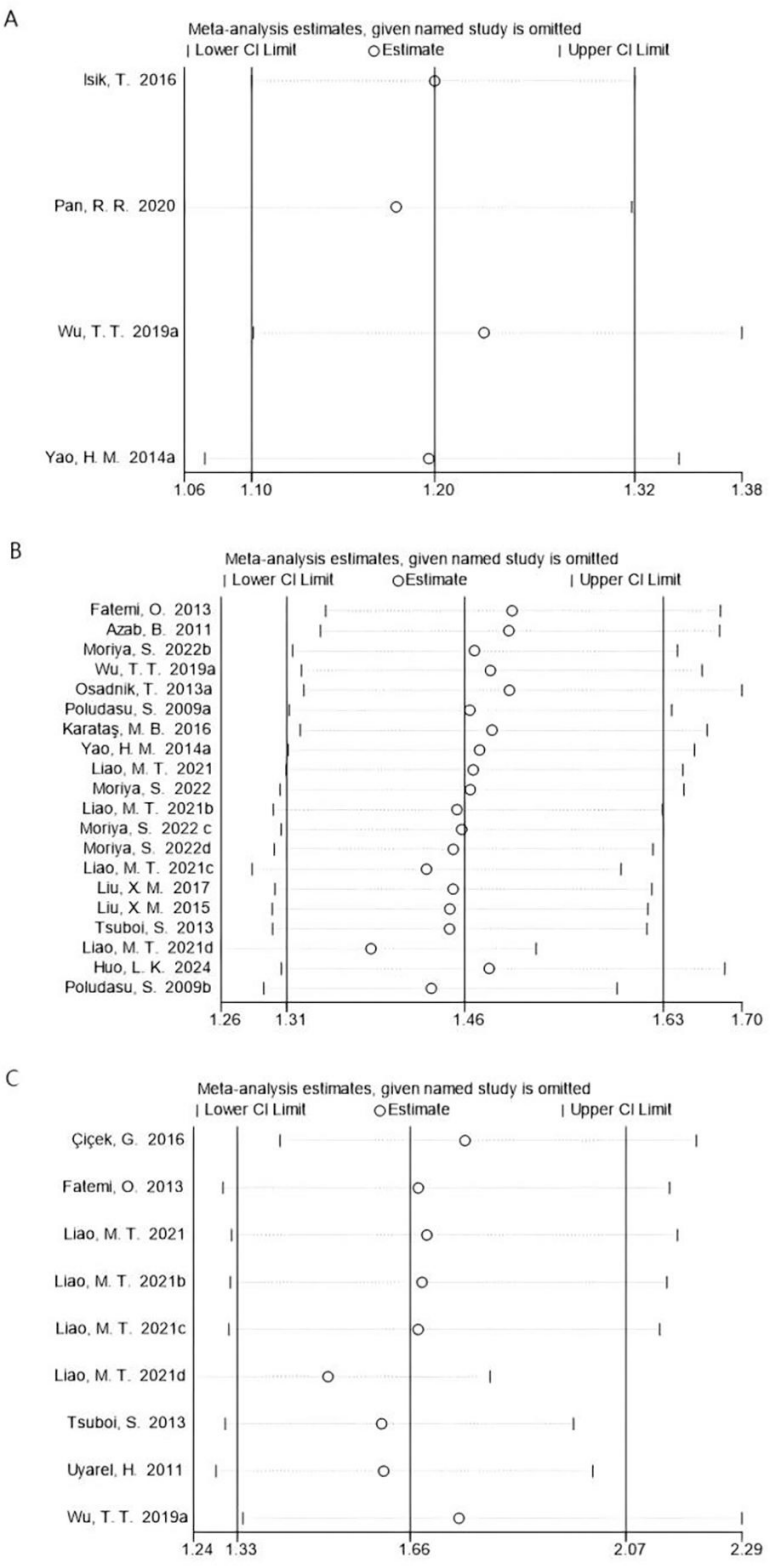
Sensitivity analyses for **(A)** standard major adverse cardiovascular events (MACE; HR-based analysis), **(B)** all-cause mortality (ACM), and **(C)** cardiovascular mortality (CVM).

Sensitivity analysis was performed only for outcomes that underwent quantitative synthesis, including HR-based standard MACE, ACM, CVM, and exploratory ISR when applicable. In the HR-based standard MACE analysis (Figure 3A), omission of the study by Pan, R. R (8)., materially changed the pooled effect estimate (revised HR = 1.27, 95% CI 0.99–1.63), indicating limited robustness and supporting cautious interpretation of this association. By contrast, sequential omission of each individual study did not materially alter the pooled estimates for ACM (Figure 3B) or CVM (Figure 3C), suggesting that these findings were relatively stable. Sensitivity analysis was additionally performed for exploratory ISR analysis when quantitative synthesis was available, and the pooled association remained statistically significant after sequential omission of each study, with all recalculated 95% confidence intervals remaining above 1. No sensitivity analysis was performed for standard MACE (OR) or isolated stent thrombosis because these outcomes were not quantitatively pooled.

### 3.5 Publication bias

**Figure 4:**
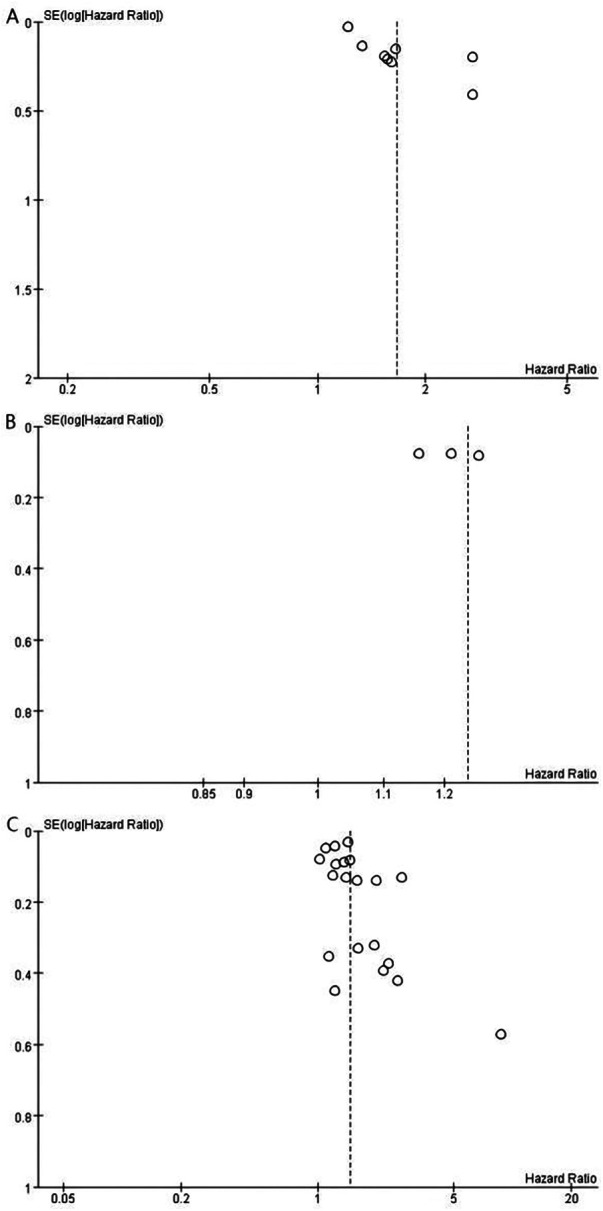
Funnel plots for the evaluation of publication bias for **(A)** cardiovascular mortality (CVM), **(B)** standard major adverse cardiovascular events (MACE; HR-based analysis) and **(C)** all-cause mortality (ACM).

Publication bias was assessed only for outcomes with a sufficient number of studies to support meaningful evaluation. For ACM, Egger’s test was used because more than 10 studies were available (*p* = 0.068), and no clear evidence of publication bias was detected; the corresponding funnel plot was visually symmetrical (Figure 4C). For HR-based standard MACE and CVM, Begg’s test was applied because of the smaller number of included studies. No clear evidence of publication bias was observed for HR-based standard MACE (*p* = 0.089), and the funnel plot appeared symmetrical (Figure 4B); however, this result should be interpreted cautiously given the limited number of studies. By contrast, Begg's test suggested potential publication bias for CVM (*p* = 0.029), and the funnel plot showed asymmetry (Figure 4A). However, trim-and-fill analysis did not materially change the pooled effect estimate (HR 1.657, 95% CI 1.327–2.070, *p* < 0.001) (Supplementary Figure S3), indicating that the overall conclusion was not substantially affected. No publication bias analysis was performed for standard MACE (OR), exploratory ISR, or isolated stent thrombosis because these outcomes were either not quantitatively pooled or included too few studies to support reliable evaluation.

## 4 Discussion

This updated meta-analysis suggests that elevated preprocedural RDW is associated with adverse outcomes after PCI, but the certainty of this association differs across endpoints. The most consistent finding was observed for all-cause mortality. Although a similar directional association was observed for cardiovascular mortality, the substantial heterogeneity indicates that this result should be interpreted cautiously. The evidence for standard MACE was less robust. After strict endpoint reclassification, only the HR-based analysis remained eligible for quantitative synthesis, and although this result was statistically significant, its robustness was limited because the association changed materially in sensitivity analysis. Accordingly, the present findings do not support RDW as an already established or uniformly applicable prognostic tool across all PCI-related outcomes.

Overall, the present findings are broadly consistent with the previous meta-analysis by Bao et al. (20). In addition, prior studies did not examine preprocedural RDW only as a mechanistic correlate of inflammation. Instead, they evaluated RDW as a routinely available baseline hematologic marker measured before PCI and assessed whether it could identify patients at higher risk of adverse outcomes after the procedure. Across DES-era CAD, stable CAD, non-ST-segment elevation acute coronary syndrome, elderly PCI cohorts, chronic coronary syndrome, and multicenter PCI populations, higher preprocedural RDW was repeatedly associated with worse long-term outcomes, particularly mortality endpoints (10, 22, 25, 26, 33, 35). Taken together, these studies suggest that preprocedural RDW may reflect baseline risk burden in patients undergoing PCI and may provide adjunctive prognostic information rather than functioning as a stand-alone established prognostic tool. The present meta-analysis further integrates these previously separate observations and provides a more outcome-specific evaluation of the prognostic relevance of preprocedural RDW.

After strict endpoint reclassification, the OR-based evidence for standard MACE was too limited for quantitative synthesis. This finding indicates that conventional composite cardiovascular endpoints should be distinguished from isolated PCI-specific stent-related outcomes. Therefore, ISR was analyzed separately as an exploratory PCI-specific outcome rather than being treated as equivalent to standard MACE. Although this exploratory analysis may provide additional information about stent-related risk, the evidence should still be interpreted cautiously because the number of eligible studies was limited and the clinical scope of the endpoint was narrower. Likewise, evidence regarding isolated stent thrombosis was limited to a single study and therefore could not be quantitatively synthesized.

Some subgroup findings may help explain between-study heterogeneity. The main relevant factors included age, clinical presentation, and RDW cutoff values. However, these subgroup findings should still be interpreted cautiously because the number of included studies was limited in several strata. Likewise, the HR-based analysis of standard MACE was unstable in sensitivity analysis, whereas both ACM and CVM showed substantial heterogeneity. Sensitivity analysis and publication bias assessment further showed that the strength of evidence differed across outcomes. No clear evidence of publication bias was observed for HR-based standard MACE. However, this result should still be interpreted cautiously because the number of included studies was limited. By contrast, potential publication bias was suggested for CVM, whereas no clear evidence of publication bias was detected for ACM. These differences may partly reflect variability in RDW cutoff definitions, study populations, endpoint definitions, and analytical adjustment strategies across the included studies. Such methodological and clinical heterogeneity may have materially influenced the pooled estimates, particularly for standard MACE and several subgroup analyses, and may partly explain why the evidence was less consistent for these outcomes than for all-cause mortality. Therefore, uncertainty should be considered explicitly when interpreting the pooled estimates, and RDW should be viewed as a risk-associated biomarker whose apparent prognostic relevance varies across outcomes and study contexts rather than as a uniformly established prognostic tool after PCI.

From a biological perspective, RDW should be interpreted as a biomarker of risk rather than as a causal factor. Elevated RDW may reflect inflammation, oxidative stress, altered erythropoiesis, and red cell dysfunction. These processes are all closely related to atherosclerosis and adverse cardiovascular risk (36–40). They may disturb iron metabolism and erythrocyte homeostasis. They may also promote vascular injury and plaque instability (36–38, 40). Therefore, RDW is better understood as an indicator of adverse biological status rather than as an established mechanistic driver of poor outcomes.

Several limitations should be considered when interpreting the present findings. First, most included studies were conducted in Asian populations. This may limit the generalizability of the pooled estimates to other regions. Second, the majority of the included studies were retrospective. Therefore, residual confounding and selection bias cannot be fully excluded. Third, RDW cutoff values were not standardized across studies. Different thresholds or derivation methods may have affected the comparability of the high-RDW and low-RDW groups and may therefore have contributed to between-study heterogeneity. In addition, the included studies enrolled clinically heterogeneous PCI populations, including ACS, CCS, STEMI, NSTEMI, stable angina, and broader CAD cohorts. These differences may have influenced the magnitude and consistency of the observed associations across outcomes. Finally, the definitions of MACE were not uniform among studies. This may have materially affected the pooled estimate for this endpoint and may partly explain why the evidence for MACE was less robust than that for ACM. Taken together, these limitations are particularly relevant to the interpretation of standard MACE and several subgroup findings, because these analyses were more sensitive to endpoint-definition variability, population heterogeneity, and limited study numbers than the ACM analysis. Therefore, although the present findings support an association between elevated RDW and adverse outcomes after PCI, the results for standard MACE, cardiovascular mortality, and several subgroup analyses should be interpreted with greater caution, whereas the association with all-cause mortality appears to be the most consistent finding.

## 5 Conclusion

In conclusion, elevated preprocedural RDW was associated with adverse outcomes after PCI. The most consistent association was observed for all-cause mortality, whereas the evidence for cardiovascular mortality and standard MACE was less certain. PCI-specific stent-related outcomes should be interpreted as exploratory. Further prospective multicenter studies with standardized endpoint definitions, more consistent analytical adjustment, and harmonized RDW cutoff values are needed to clarify the clinical relevance of RDW in this setting.

## Data Availability

The original contributions presented in the study are included in the article/Supplementary Material, further inquiries can be directed to the corresponding authors.

## Supplementary material

Supplementary Figure S1 Forest plot for the exploratory association between preprocedural red cell distribution width (RDW) and in-stent restenosis (ISR).

Supplementary Figure S2 Sensitivity analysis for the exploratory association between preprocedural red cell distribution width (RDW) and in-stent restenosis (ISR).

Supplementary Figure S3 Trim-and-fill analysis for cardiovascular mortality (CVM).

Supplementary Table S1 The detailed search strategy.

Supplementary Table S2 Quality evaluation of the eligible studies with Newcastle-Ottawa scale.

Supplementary Table S3 Handling of overlapping cohorts.
