# Supplementary material for: Prognostic value of red cell distribution width in patients undergoing percutaneous coronary intervention: a systematic review and meta-analysis
Source: Front Cardiovasc Med. 2026 Jul 22;13:1787857. doi: 10.3389/fcvm.2026.1787857 (PMC13437508; doi:10.3389/fcvm.2026.1787857)
Supplement: Supplementary file 1 [file Table1.docx]

PubMed-98

**((("Erythrocyte Indices"[Mesh]) OR ((((((((((((((((((((Red Cell Indexes) OR (Erythrocyte Index)) OR (Erythrocyte Indexes)) OR (Red Cell Index)) OR (Red Cell Indices)) OR (Erythrocyte Size Determination)) OR (Erythrocyte Size Determinations)) OR (Mean Corpuscular Volume)) OR (Mean Corpuscular Volumes)) OR (Erythrocyte Volume, Mean Cell)) OR (Mean Cell Volume)) OR (Mean Cell Volumes)) OR (Mean Corpuscular Hemoglobin)) OR (Mean Corpuscular Hemoglobins)) OR (Erythrocyte Thickness)) OR (Erythrocyte Diameter)) OR (Erythrocyte Diameters)) OR (Mean Corpuscular Hemoglobulin Concentration)) OR (Mean Cell Hemoglobin Concentration)) OR (Red Cell Distribution Width))) AND (("Coronary Artery Disease"[Mesh]) OR (((((((((Coronary Artery Diseases) OR (Coronary Arteriosclerosis)) OR (Coronary Arterioscleroses)) OR (Coronary Atherosclerosis)) OR (Coronary Atheroscleroses)) OR (Left Main Coronary Artery Disease)) OR (Left Main Coronary Disease)) OR (Lft Main Disease)) OR (Left Main Diseases)))) AND (("Percutaneous Coronary Intervention"[Mesh]) OR (((Prcutaneous Coronary Interventions) OR (Percutaneous Coronary Revascularization)) OR (Percutaneous Coronary Revascularizations)))**

Embase-80

((Erythrocyte Indices or (Red Cell Indexes or Erythrocyte Index or Erythrocyte Indexes or Red Cell Index or Red Cell Indices or Erythrocyte Size Determination or Erythrocyte Size Determinations or Mean Corpuscular Volume or Mean Corpuscular Volumes or Erythrocyte Volume, Mean Cell or Mean Cell Volume or Mean Cell Volumes or Mean Corpuscular Hemoglobin or Mean Corpuscular Hemoglobins or Erythrocyte Thickness or Erythrocyte Diameter or Erythrocyte Diameters or Mean Corpuscular Hemoglobulin Concentration or Mean Cell Hemoglobin Concentration or Red Cell Distribution Width)) and (Coronary Artery Disease or (Coronary Artery Diseases or Coronary Arteriosclerosis or Coronary Arterioscleroses or Coronary Atherosclerosis or Coronary Atheroscleroses or Left Main Coronary Artery Disease or Left Main Coronary Disease or Lft Main Disease or Left Main Diseases)) and (Percutaneous Coronary Intervention or (Prcutaneous Coronary Interventions or Percutaneous Coronary Revascularization or Percutaneous Coronary Revascularizations))).af.

Cochrane-1

((Erythrocyte Indices or (Red Cell Indexes or Erythrocyte Index or Erythrocyte Indexes or Red Cell Index or Red Cell Indices or Erythrocyte Size Determination or Erythrocyte Size Determinations or Mean Corpuscular Volume or Mean Corpuscular Volumes or Erythrocyte Volume, Mean Cell or Mean Cell Volume or Mean Cell Volumes or Mean Corpuscular Hemoglobin or Mean Corpuscular Hemoglobins or Erythrocyte Thickness or Erythrocyte Diameter or Erythrocyte Diameters or Mean Corpuscular Hemoglobulin Concentration or Mean Cell Hemoglobin Concentration or Red Cell Distribution Width)) and (Coronary Artery Disease or (Coronary Artery Diseases or Coronary Arteriosclerosis or Coronary Arterioscleroses or Coronary Atherosclerosis or Coronary Atheroscleroses or Left Main Coronary Artery Disease or Left Main Coronary Disease or Lft Main Disease or Left Main Diseases)) and (Percutaneous Coronary Intervention or (Prcutaneous Coronary Interventions or Percutaneous Coronary Revascularization or Percutaneous Coronary Revascularizations))).af.

WOS-109

(((Erythrocyte Indices) OR ((((((((((((((((((((Red Cell Indexes) OR (Erythrocyte Index)) OR (Erythrocyte Indexes)) OR (Red Cell Index)) OR (Red Cell Indices)) OR (Erythrocyte Size Determination)) OR (Erythrocyte Size Determinations)) OR (Mean Corpuscular Volume)) OR (Mean Corpuscular Volumes)) OR (Erythrocyte Volume, Mean Cell)) OR (Mean Cell Volume)) OR (Mean Cell Volumes)) OR (Mean Corpuscular Hemoglobin)) OR (Mean Corpuscular Hemoglobins)) OR (Erythrocyte Thickness)) OR (Erythrocyte Diameter)) OR (Erythrocyte Diameters)) OR (Mean Corpuscular Hemoglobulin Concentration)) OR (Mean Cell Hemoglobin Concentration)) OR (Red Cell Distribution Width))) AND ((Coronary Artery Disease) OR (((((((((Coronary Artery Diseases) OR (Coronary Arteriosclerosis)) OR (Coronary arteriosclerosis)) OR (Coronary Atherosclerosis)) OR (Coronary atherosclerosis)) OR (Left Main Coronary Artery Disease)) OR (Left Main Coronary Disease)) OR (Lft Main Disease)) OR (Left Main Diseases)))) AND ((Percutaneous Coronary Intervention) OR (((percutaneous Coronary Interventions) OR (Percutaneous Coronary Revascularization)) OR (Percutaneous Coronary Revascularizations))) (Topic)
