# Supplementary material for: Prognostic value of red cell distribution width in patients undergoing percutaneous coronary intervention: a systematic review and meta-analysis
Source: Front Cardiovasc Med. 2026 Jul 22;13:1787857. doi: 10.3389/fcvm.2026.1787857 (PMC13437508; doi:10.3389/fcvm.2026.1787857)
Supplement: Supplementary file 2 [file Table2.docx]

| Supplementary Table S2. Quality evaluation of the eligible studies with Newcastle–Ottawa scale. | | | | | | | | | |
| --- | --- | --- | --- | --- | --- | --- | --- | --- | --- |
| Study | Selection | | | | Comparability | | Outcome | | |
|  | Representative-ness | Selection of  non-exposed | Ascertainment  of exposure | Outcome not present at start | Comparability on most important factors | Comparability on other risk factors | Assessment of outcome | Long enough follow-up (median≥1 year) | Adequacy  (completeness) of follow-up |
| Tunçez, A. 2017 | * | * | * | * | - | - | * | * | * |
| Karataş, M. B. 2016 | * | * | * | * | - | - | * | * | * |
| Huo, L. K. 2024 | * | * | * | * | - | - | * | * | * |
| Li, C. 2019 | * | * | * | * | - | - | * | * | * |
| Isik, T. 2016 | * | * | * | * | - | - | * | * | * |
| Tsuboi, S. 2013 | * | * | * | * | - | - | * | * | * |
| Liu, X. M. 2017 | * | * | * | * | - | * | * | * | * |
| Zhuo, M. F. 2022 | * | * | * | * | - | - | * | * | * |
| Zhou, J. 2022 | - | * | * | * | * | * | * | * | * |
| Yildiz, A. 2014 | * | * | * | * | - | - | * | * | * |
| Yao, H. M. 2014 | * | * | * | * | - | - | * | * | * |
| Wu, T. T. 2019 | * | * | * | * | - | - | * | * | * |
| Poludasu, S. 2009 | - | * | * | * | - | - | * | * | * |
| Liao, M. T. 2021 | * | * | * | * | - | - | * | * | * |
| Uyarel, H. 2011 | * | * | * | * | - | - | * | * | * |
| Fatemi, O. Paranilam, J.  2013 | - | * | * | * | - | - | * | * | * |
| Osadnik, T. 2013 | * | * | * | * | - | - | * | * | * |
| Moriya, S. 2022 | * | * | * | * | - | - | * | * | * |
| Liu, X. M. 2015 | - | * | * | * | - | * | * | * | * |
| Pan, R. R. 2020 | * | * | * | * | - | - | * | * | * |
| Zhao, K. 2015 | * | * | * | * | - | * | * | * | * |
| Ösken, A. 2024 | * | * | * | * | - | - | * | * | * |
| Azab, B. 2011 | * | * | * | * | - | - | * | * | * |
| Çiçek, G. 2016 | * | * | * | * | - | - | * | * | * |
| *indicates criterion met; - indicates significant of criterion not met. | | | | | | | | | |
