# Supplementary material for: Prognostic value of red cell distribution width in patients undergoing percutaneous coronary intervention: a systematic review and meta-analysis
Source: Front Cardiovasc Med. 2026 Jul 22;13:1787857. doi: 10.3389/fcvm.2026.1787857 (PMC13437508; doi:10.3389/fcvm.2026.1787857)
Supplement: Supplementary file 3 [file Table3.docx]

| Author | study period | region | Center / hospital | Population | MACE | MACEs definition | All-­cause mortality | Cardiovascular mortality | ISR / stent-related outcome | ISR / stent-related outcome definition | Overlap assessment |
| --- | --- | --- | --- | --- | --- | --- | --- | --- | --- | --- | --- |
| Tunçez, A. 2017 | 2009-2013 | Turkey | high-volume tertiary center；exact recruiting hospital not explicitly stated in the main text | STEMI | NA | NA | NA | NA | OR:1.397,  95%CI:1.177-1.657 | Stent throm bosis | No apparent overlap |
| Karataş, M. B. 2016 | 2009-2013 | Turkey | Dr. Siyami Ersek Training and Research Hospital | STEMI | NA | NA | HR:1.24,  95% CI: 1.03-1.47 | NA | NA | NA | No apparent overlap |
| Yildiz, A. 2014 | 2008-2013 | Turkey | Istanbul Medipol Hospital / Istanbul Medipol University, Istanbul, Turkey | CAD | NA | NA | NA | NA | OR :1.4, 95%CI:1.18-1.67 | BMS-ISR | No apparent overlap |
| Çiçek, G. 2016 | 2010-2015 | Turkey | Staten Island University Hospital, Staten Island, New York, USA | STEMI | NA | cardiovascular death + re-infarction + TVR | NA | HR:1.213;  95%CI:1.138-1.291 | NA | NA | No apparent overlap |
| Isik, T. 2016 | 2010-2010 | Turkey | exact recruiting hospital not explicitly stated in the main text， authors come from Balikesir University、Mustafa Kemal University、Ataturk University、Ordu University、Bezmialem University | STEMI | HR: 5.26，  95% CI: 1.71-6.1 | MACE were defined as cardiovascular mortality, repeat target vessel revascularization (TVR), and reinfarction (re-MI).no stroke | NA | NA | NA | NA | No apparent overlap |
| Ösken, A. 2024 | 2015-2017 | Turkey | Health Sciences University Dr. Siyami Ersek Thoracic and Cardiovascular Surgery Center, Istanbul, Turkey | ACS | NA | NA | NA | NA | OR:1.183; 95%CI:1.034-1.353 | ISR | No apparent overlap |
| Uyarel, H. 2011 | 2003-2008 | Turkey | Siyami Ersek Cardiovascular and Thoracic Surgery Center, Istanbul, Turkey | STEMI | NA | cardiovascular mortality + reinfarction + repeat target-vessel revascularization (PCI or surgery) | NA | HR:2.703;  95%CI:1.208-6.048 | NA | NA | No apparent overlap |
| Huo, L. K. 2024 | 2019-2022 | China | The Second Hospital of Tianjin Medical University | CAD | NA | NA | HR: 1.437,  95%CI1.346-1.535 | NA | NA | NA | No apparent overlap |
| Zhao, K. 2015 | 2010-2014 | China | Tianjin Nankai Hospital, Tianjin, China | stable angina  pectoris (SAP) | NA | NA | NA | NA | OR:1.4; 95%CI:1.18-1.67 | DES-ISR | No apparent overlap |
| Li, C. 2019 | 2010-2013 | China | Zhongshan Hospital, Fudan University, Shanghai Institute of Cardiovascular Diseases, Shanghai, China | CTO | NA | NA | NA | NA | OR:1.324,, 95%CI:1.134-1.545 | ISR | No apparent overlap |
| Liu, X. M. 2015 | 2009-2011 | China | Beijing Anzhen Hospital, Capital Medical University, Beijing, China | ≥65 years' CAD | NA | NA | HR:2.301;  95%CI:1.106-4.785 | NA | NA | NA | Different Population：Liu,X,M.2015:≥65 years' CAD |
| Liu, X. M. 2017 | 2009-2011 | China | Beijing Anzhen Hospital, Capital Medical University, Beijing, China | NSTEACS | NA | NA | HR:2.171, 95%:CI :1.007-4.680 | NA | NA | NA | Different Population：Liu,X,M.2017:NSTEACS |
| Zhuo, M. F. 2022 | 2018-2020 | China | Shishi General Hospital, Fujian Province, China | acute myocardial infarction with high throm botic load | OR1.781,  95%CI:1.157-2.741 | recurrent angina pectoris + recurrent acute MI + sudden cardiac death + malignant arrhythmia + congestive heart failure + other cardiovascular events | NA | NA | NA | NA | No apparent overlap |
| Zhou, J. 2022 | 2016-2018 | two centers | Two-center study: Taicang Hospital + Zhongshan Hospital (Fudan University) | ACS | NA | NA | NA | NA | OR :1.54, 95%CI:1.14-2.08 | ISR | No apparent overlap |
| Liao, M. T. 2021a | 2006-2017 | multi-center | Multicenter: NTUH system (Taipei / Yunlin / Hsinchu branches) | CAD | NA | NA | HR:1.394;  95%CI1.078-1.804 | HR:1.533;  95% CI 1.049-2.240 | NA | NA | No apparent overlap |
| Liao, M. T. 2021b | 2006-2017 | multi-center | Multicenter: NTUH system (Taipei / Yunlin / Hsinchu branches) | CAD | NA | NA | HR:1.592;  95%CI1.208-2.099 | HR:1.568;  95% CI 1.029-2.387 | NA | NA | No apparent overlap |
| Liao, M. T. 2021c | 2006-2017 | multi-center | Multicenter: NTUH system (Taipei / Yunlin / Hsinchu branches) | CAD | NA | NA | HR:2.003;  95%CI1.518-2.643 | HR:1.609;  95% CI 1.031-2.509 | NA | NA | No apparent overlap |
| Liao, M. T. 2021d | 2006-2017 | multi-center | Multicenter: NTUH system (Taipei / Yunlin / Hsinchu branches) | CAD | NA | NA | HR:2.689;  95%CI2.076-3.485 | HR:2.710;  95% CI:1.825-4.026 | NA | NA | No apparent overlap |
| Yao, H. M. 2014 | 2009-2011 | China | The First Affiliated Hospital of Zhengzhou University, Zhengzhou, China | CAD | HR：1.21;  95% CI：1.04-1.39 | Death+MI+stroke | HR:1.37;  95% CI : 1.15-1.62 | NA | NA | NA | No apparent overlap |
| Pan, R. R. 2020 | 2016-2018 | China | Affiliated Hospital of Nantong University, Nantong, Jiangsu Province, China | ACS | HR:1.258; 95%CI:1.066-1.363 | cardiac death + recurrent MI + CHF + atherosclerotic heart disease + hypoxic encephalopathy + fatal/nonfatal ischemic stroke | NA | NA | NA | NA | No apparent overlap |
| Wu, T. T. 2019 | 2008-2016 | China | First Affiliated Hospital of Xinjiang Medical University, Urumqi, China | CAD | HR：1.155;  95% CI0.994-1.341 | cardiac death + recurrent MI + TVR | HR:1.203;  95%CI:0.941-1.57 | HR:1.331;  95% CI:1.009-1.755 | NA | NA | No apparent overlap |
| Azab, B. 2011 | 2004-2006 | United States | Staten Island University Hospital, Staten Island, New York, USA | NSTEMI | NA | NA | HR:1.104;  95%CI:1.004-1.23 | NA | NA | NA | No apparent overlap |
| Poludasu, S. 2009a | 2003-2005 | United States | SUNY Downstate Medical Center, New York, USA | STEMI | NA | NA | HR:1.23;  95%CI: 0.51-2.96 | NA | NA | NA | No apparent overlap |
| Poludasu, S. 2009b | 2003-2005 | United States | SUNY Downstate Medical Center, New York, USA | STEMI | NA | NA | HR:8.65;  95%CI:2.82-26.56 | NA | NA | NA | No apparent overlap |
| Fatemi, O. Paranilam, J.2013 | 2004-2007 | four centers | Four-center EVENT substudy (USA) | CAD | NA | NA | HR:1.03;  95%CI:0.88-1.21 | HR:1.65,;  95%CI:1.22-2.23 | NA | NA | No apparent overlap |
| Osadnik, T. 2013 | 2007-2011 | Poland | Silesian Center for Heart Diseases, Zabrze, Poland | Stable coronaryartery disease | NA | NA | HR:1.23;  95% CI:1.13-1.35 | NA | NA | NA | No apparent overlap |
| Moriya, S. 2022a | 2002-2016 | Japan | Juntendo University hospital system, Japan | CCS | NA | NA | HR:1.46;  95%CI:1.24-1.69 | NA | NA | NA | No apparent overlap |
| Moriya, S. 2022b | 2002-2016 | Japan | Juntendo University hospital system, Japan | CCS | NA | NA | HR:1.14;  95%CI:0.57-2.27 | NA | NA | NA | No apparent overlap |
| Moriya, S. 2022c | 2002-2016 | Japan | Juntendo University hospital system, Japan | CCS | NA | NA | HR:1.62;  95%CI:0.85-3.11 | NA | NA | NA | No apparent overlap |
| Moriya, S. 2022d | 2002-2016 | Japan | Juntendo University hospital system, Japan | CCS | NA | NA | HR:1.95;  95%CI:1.04-3.67 | NA | NA | NA | No apparent overlap |
| Tsuboi, S. 2013 | 2000-2007 | Japan | Juntendo University School of Medicine, Tokyo, Japan | Stable CAD | NA | NA | HR:2.56;  95%CI:1.12-6.62 | HR:16.2;  95% CI,:2.09-124.7 | NA | NA | No apparent overlap |
